# Digital Rehabilitation for Elbow Pain Musculoskeletal Conditions: A Prospective Longitudinal Cohort Study

**DOI:** 10.3390/ijerph19159198

**Published:** 2022-07-27

**Authors:** Dora Janela, Fabíola Costa, Maria Molinos, Robert G. Moulder, Jorge Lains, Virgílio Bento, Justin K. Scheer, Vijay Yanamadala, Steven P. Cohen, Fernando Dias Correia

**Affiliations:** 1 SWORD Health, Inc., Draper, UT 84043, USA; d.janela@swordhealth.com (D.J.); f.costa@swordhealth.com (F.C.); mmolinos@swordhealth.com (M.M.); vbento@swordhealth.com (V.B.); v.yanamadala@swordhealth.com (V.Y.); 2 Institute for Cognitive Science, University of Colorado Boulder, Boulder, CO 80309, USA; robertgm111@gmail.com; 3 Rovisco Pais Medical and Rehabilitation Centre, 3064-908 Tocha, Portugal; jorgelains@sapo.pt; 4 Faculty of Medicine, Coimbra University, 3004-504 Coimbra, Portugal; 5 Department of Neurological Surgery, University of California, San Francisco, CA 94143, USA; justin.scheer@ucsf.edu; 6 Department of Surgery, Frank H. Netter School of Medicine, Quinnipiac University, Hamden, CT 06473, USA; 7 Department of Neurosurgery, Hartford Healthcare Medical Group, Westport, CT 06103, USA; 8 Department of Anesthesiology & Critical Care Medicine, Johns Hopkins School of Medicine, Baltimore, MD 21287, USA; scohen40@jhmi.edu; 9 Department of Physical Medicine and Rehabilitation, Johns Hopkins School of Medicine, Baltimore, MD 21287, USA; 10 Department of Neurology, Johns Hopkins School of Medicine, Baltimore, MD 21287, USA; 11 Department of Psychiatry and Behavioral Sciences, Johns Hopkins School of Medicine, Baltimore, MD 21287, USA; 12 Department of Anesthesiology, Uniformed Services University of the Health Sciences, Bethesda, MD 20814, USA; 13 Department of Physical Medicine and Rehabilitation, Uniformed Services University of the Health Sciences, Bethesda, MD 20814, USA; 14 Department of Neurology, Centro Hospitalar e Universitário do Porto, 4099-001 Porto, Portugal

**Keywords:** musculoskeletal pain, physical therapy, telerehabilitation, digital therapy, eHealth, motion trackers

## Abstract

Elbow musculoskeletal pain (EP) is a major cause of disability. Telerehabilitation has shown great potential in mitigating musculoskeletal pain conditions, but EP is less explored. This single-arm interventional study investigates clinical outcomes and engagement levels of a completely remote multimodal digital care program (DCP) in patients with EP. The DCP consisted of exercise, education, and cognitive-behavioral therapy for 8 weeks. Primary outcome: disability change (through the Quick Disabilities of the Arm, Shoulder, and Hand questionnaire (QuickDASH), treatment response cut-offs: 12.0-point reduction and 30% change). Secondary outcomes: pain, analgesic intake, surgery intent, mental health, fear–avoidance beliefs, work productivity, and patient engagement. Of the 132 individuals that started the DCP, 112 (84.8%) completed the intervention. Significant improvements were observed in QuickDASH with an average reduction of 48.7% (11.9, 95% CI 9.8; 14.0), with 75.3% of participants reporting ≥30% change and 47.7% reporting ≥12.0 points. Disability change was accompanied by reductions in pain (53.1%), surgery intent (57.5%), anxiety (59.8%), depression (68.9%), fear–avoidance beliefs (34.2%), and productivity impairment (72.3%). Engagement (3.5 (SD 1.4) sessions per week) and satisfaction 8.5/10 (SD 1.6) were high. The significant improvement observed in clinical outcomes, alongside high engagement, and satisfaction suggests patient acceptance of this care delivery mode.

## 1. Introduction

Upper extremity musculoskeletal (MSK) conditions are a major cause of reduced ability to work [1]. Within these, elbow pain (EP) and particularly elbow tendinopathies are highly prevalent, accounting for approximately 20% of work-related MSK conditions [2], with nearly one million new cases each year in the United States (US) [3]. With such a large incidence of elbow tendinopathies, there is a substantial socioeconomic impact resulting from productivity loss, sick leave benefits, and healthcare utilization [4,5,6,7]. EP can be accompanied by other symptoms, namely radiating pain, paresthesia, stiffness, and instability, comprising movement activities, especially involving push, pull, and grip [8].

The pathophysiological mechanisms of EP are still not fully understood; however, etiology is known to be multifactorial. Psychosocial status [9,10], lifestyle [11,12], and occupational demands (such as heavy load handling and highly repetitive movements with the wrist/hand) [11,13,14,15,16] are important risk factors. Further, these conditions can also affect mental health [17]. Despite a lack of strong evidence regarding optimal management, the general consensus has been to prioritize conservative interventions [18,19] and restrict surgery to the small proportion of patients who have failed conservative care [3,4,19,20]. Corticosteroid injections are commonly used; however, long-term results are inconsistent [19,21,22]. In most cases, exercise-based interventions are effective at reducing pain and improving function, with superior results compared to other passive interventions such as ultrasound and friction massage [18,19,21,22,23]. With the increasing burden of MSK disease [24] and recent limitations imposed by the COVID-19 pandemic, proper and timely treatment may not always be accessible [25,26], which has fostered an interest in novel care delivery systems. Telerehabilitation, using technology to provide healthcare, has shown consistently positive results in the recovery of several MSK conditions [27,28,29,30,31]. Accessibility is enhanced by reducing travel, geographic, and time barriers, while helping to ease the financial burden [32,33]. On the other hand, telemedicine might be the missing tool for healthcare providers to tackle practitioners’ shortages, facilities’ uneven distribution, and improve care cost-efficiency [34,35]. Digital interventions have the opportunity to increase the quality of care through integrated evidence-based treatments [1,33,36]. Its convenience also promotes patient adherence and self-management [37].

Previous studies, analyzing the effectiveness of telerehabilitation in other MSK conditions, show similar or even superior results in comparison to face-to-face care [28,38]. Thus, telerehabilitation approaches are currently being explored for EP assessment [39] and management [40]. However, studies evaluating telehealth for EP are still scarce, and none focus on non-specific EP [27,28,41], prompting the need for further research. Nevertheless, a pilot study reporting similar outcomes between in-person care and a hybrid format of telerehabilitation hinted that digital interventions for EP management may have similar outcomes as conventional therapies [40].

Previously, we have demonstrated the utility of a completely remote digital care program (DCP) integrating exercise, education, and cognitive behavioral therapy (CBT) in several MSK conditions [42,43,44,45], including for upper limb [46,47,48]. The present study seeks to investigate the clinical outcomes and engagement of this DCP in a real-world cohort of patients with EP. We hypothesized that the EP-related outcomes changes would be improved after the intervention.

## 2. Materials and Methods

### 2.1. Study Design

This is a single-arm, decentralized study evaluating participants with EP receiving a completely remote, multimodal digital care program (DCP). Consecutive participants who participated in the DCP until the cut-off date of October 20, 2021 were eligible for enrollment. Informed consent was provided by all participants before enrollment. The home-based DCP was delivered between 24 June 2020 and 12 January 2022.

This prospective cohort study was approved by the New England Institutional Review Board (number 120190313) with registration occurring on ClinicalTrials.gov (NCT04092946) on 17 September 2019.

### 2.2. Participants

Adults (>18 years) with self-reported EP complaints, and beneficiaries of health plans of employers, who applied to the SWORD Health DCP, were invited to participate in the study through a screening questionnaire on a dedicated website. Exclusion criteria were: (1) suspected serious injury with the inability to actively move the affected segment; (2) serious injury not cleared for exercise by the attending physician; (3) the presence of a health condition (e.g., cardiac, respiratory, or other) incompatible with at least 20 min of light to moderate exercise; (4) undergoing treatment for cancer; (5) rapidly progressive loss of strength and/or numbness in the affected arm; and (6) ability to understand simple and complex motor commands.

### 2.3. Intervention

**Figure 1:**
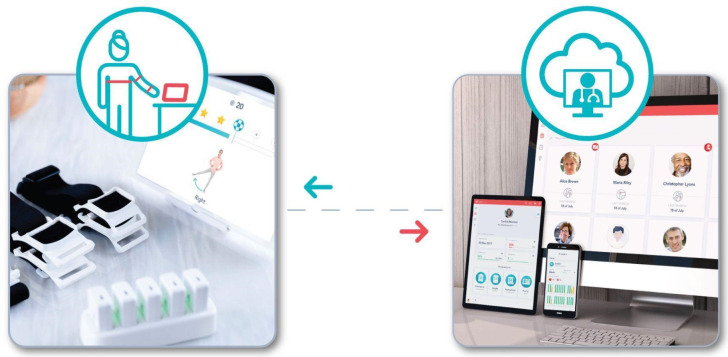
System components. The left figure shows the motion tracker setup and the mobile app displaying the audio-video instructions during the exercise, alongside the real-time biofeedback provided to patients. The right figure depicts the web portal with the results from each patient’s session, enabling fully remote asynchronous monitoring by the assigned physical therapist.

After enrollment, each patient was followed by an assigned physical therapist (PT) until the end of the 8-week intervention. The PT was responsible for evaluating the participant’s condition through anamnesis and physical exam. In the presence or suspicion of signs and symptoms indicators of possible serious pathology (red flags), the PT directs the participant to a physician who screens and confirms eligibility. The DCP incorporated exercise, education, and cognitive behavioral therapy (CBT). Exercise programs consisted of gradual progressive movement exposure prescribed by the assigned PT who adjusted the program according to patient needs and progress. Participants were advised to complete at least 4 exercise sessions per week performed through an FDA-listed medical device. The medical device is comprised of inertial motion trackers (IMU), a mobile app integrated on a dedicated tablet, and a cloud-based portal (Figure 1).

The tablet displayed the exercise sessions and provided real-time biofeedback through audio-video cues based on motion digitized through IMUs (placed on the chest, arm, and forearm using straps), allowing participants to perform sessions independently at their convenience. Data were collected and stored in a web-based portal, enabling complete remote asynchronous monitoring by the PT. Participants were still considered if they were compliant with the intervention but failed to complete a given reassessment survey. Participants who did not perform an exercise session for 28 consecutive days were considered dropouts.

Participants and PTs were able to communicate regularly through videocalls or built-in secure chats within the mobile app, at least once per week. Education was delivered through short articles, as well as through pre-recorded audio sessions and interactive modules based on CBT. Content targeted pathoanatomy mechanisms, the relevance of physical activity and exercise, activity pacing/modification, and self-management. The CBT program, created by a multidisciplinary team including psychiatrists and psychologists, was based on third-generation CBT techniques–mindfulness, acceptance and commitment therapy, and empathy-focused therapy, to address fear–avoidance, pain reconceptualization, and active coping skills. These components focused on empowering patients about their condition and providing self-management skills.

### 2.4. Outcomes

Assessments were conducted at baseline, 4- and 8-weeks. Change trajectories were modeled through latent growth curve analysis (LGCA) (see Statistical Analyses section) using all time points which allowed the estimation of changes between baseline and 8 weeks for each outcome.

The primary outcome was patient-reported disability through the Quick Disabilities of the Arm, Shoulder, and Hand questionnaire (QuickDASH), an 11-item questionnaire with a Likert scale addressing disability and symptom severity, which has been demonstrated to be valid for assessing elbow conditions [49]. Scores range from 0 to 100% (higher scores indicating poorer functioning) [50]. To assess how many participants responded to the intervention, a literature search was conducted for the elbow-related QuickDASH minimal clinically important changes (MCIC). Reports were mainly focused on generic upper extremity conditions cohorts with high baseline disability. To account for possible flooring effects, response to treatment was evaluated considering both an absolute MCIC of 12 points [51,52], and also a relative 30% mean change threshold as suggested in prior upper limb research [53,54]. Any patient that met either of those thresholds was considered a responder.

Secondary outcomes comprised the following domains (in all scales higher scores relate to worse condition):

- Pain was assessed through an 11-point numerical pain rating scale (NPRS) through the question, “Please rate your average pain over the last 7 days: 0 (no pain at all) to 10 (worst pain imaginable)” [55];
- Analgesic use through the question: “Are you currently taking any pain medication?”;
- Self-reported surgery intent through the question, “How likely are you to seek surgery to address your condition in the next 12 months?” (range 0–100);
- Mental health including anxiety and depression levels through the Generalized Anxiety Disorder 7-item scale (GAD-7) (scores 0–21) [56,57] and Patient Health 9-item questionnaire (PHQ-9) (scores 0–27) [56,58], respectively. A cut-off threshold of ≥5 indicates at least mild anxiety/depression [59]. Fear-avoidance beliefs (FAB) were also assessed, through the 5-item questionnaire for physical activity (FABQ-PA), scored from 0 to 24 [60,61];
- Work productivity in employed participants through the Work Productivity and Activity Impairment questionnaire for general health (WPAI), assessed by the sub-scores: WPAI overall (combining presenteeism and absenteeism), WPAI work (presenteeism), WPAI time (absenteeism), and WPAI activities (activities of daily living impairment) (scores 0–100%) [62];
- Patient engagement of DCP was measured by completion of the program (completion rate), the number of completed exercise sessions, and the number of sessions performed per week;
- Overall satisfaction by the question: “On a scale from 0 to 10, how likely is it that you would recommend this intervention to a friend or neighbor?”.

### 2.5. Safety and Adverse Events

Participants were advised to report pain and fatigue levels at the end of each exercise session on a 0–10 NRS, as well as to report any adverse event (e.g., worsening of symptomatology, the appearance of new signs or symptoms, or other events that could interfere with the condition or the execution of the intervention) to the dedicated PT through the available communication channels for further assessment.

### 2.6. Data Availability

All relevant data are included in the article or available as Supplementary Materials. De-identified data and analysis codes are available upon reasonable request to the corresponding author.

### 2.7. Statistical Analyses

Descriptive statistics were applied to characterize the study population and engagement metrics, with continuous variables reported as mean (standard deviation) and categorical variables as frequencies (percentage). Differences in baseline characteristics between completers and non-completers (participants that were excluded or dropped out after starting the program) and between different engagement levels were assessed through chi-squared tests for categorical variables and independent samples *t*-tests for continuous variables.

For longitudinal data analysis, LGCA was applied, a methodology from the same family of linear mixed-effects modeling but estimated as a structural equation model (see Supplementary Figure S1) [63], with the advantages of providing a measure of model fitness, and allowing the use of full information maximum likelihood (FIML) to address missing data, which outperforms other modern imputation models such as multiple imputations by chained equations (MICE) and listwise deletion [64,65]. FIML estimation considers all available data at each time point from all participants [66,67,68,69].

LGCA estimates outcomes trajectories over time, based on the individual trajectories and considering time as a continuous variable. This provides an estimate of the average trajectory, and individual variation around that trajectory over time [68,69]. Trajectories were calculated through intercept (i.e., initial estimated value at baseline) and slope (i.e., linear outcome change per week) for each variable. Analyses followed an intent-to-treat approach, considering all participants and additionally filtering for clinically significant scores at baseline, i.e., surgery intent and WPAI > 0 points, GAD-7, and PHQ-9 ≥ 5 points. In addition, a conditional analysis was conducted to assess the influence of age, sex, body mass index (BMI), and acuity as covariates. Models were adjusted for these covariates and controlled for early discharge, fitted as random effects allowing each to vary between individuals. All models were estimated with a robust sandwich estimator for standard errors.

Model fitness was assessed through chi-squared test, root mean square error of approximation (RMSEA), confirmatory fit index (CFI), and standardized root mean square residual (SRMR), using the following cut-off criteria: CFI = close to 0.95; RMSEA = close to 0.06 and SRMR = close to 0.08 [70,71].

A logistic regression analysis was performed to calculate the odds ratio (OR) for being a responder considering the primary outcome, as well as the effect of age, BMI, sex, and acuity covariates.

Bivariate correlations (Pearson r) were used to investigate associations between outcome changes. Significance levels were considered as *p* < 0.05 in all analyses. LGCA was coded using R (version 1.4.1717) and all other analyses using SPSS (version 17.0, SPSS Inc, Chicago, IL, USA).

## 3. Results

### 3.1. Participants

**Figure 2:**
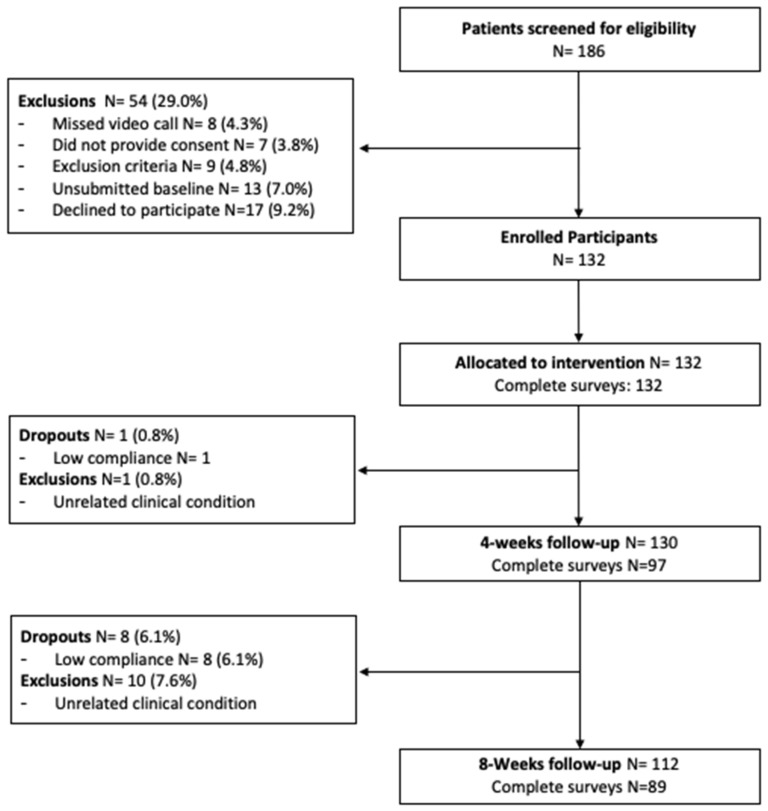
Study flow diagram.

A total of 186 participants were screened for eligibility (Figure 2). Of these, 54 were excluded. In total, 132 participants from 32 states in the US started the program, of which 112 completed the study (84.8% completion rate). Baseline demographic characteristics (N = 132) are presented in Table 1.

The average participant age was 51.3 (9.9) years, with most being female (60.8%) and employed (93.2%). No significant baseline differences were observed between completers (N = 112) and non-completers (N = 20) (Supplementary Table S1), except for the completers having a higher percentage of individuals with lateral elbow tendinopathies and injury-related EP (*p* = 0.014), higher PHQ-9 scores (2.0 (3.4) vs. 1.2 (1.5)) (*p* = 0.041) and higher WPAI overall at baseline (12.2 (20.4) vs. 3.4 (7.5)) (*p* < 0.001).

### 3.2. Clinical Outcomes

Outcomes LGCA unconditional modeling (Supplementary Table S2) allowed for the estimation of each outcome average change as reported at Table 2. These results, alongside the impact of covariates on each outcome (Supplementary Table S3), are discussed in the following subsections.

#### 3.2.1. Primary Outcome

##### QuickDASH

**Figure 3:**
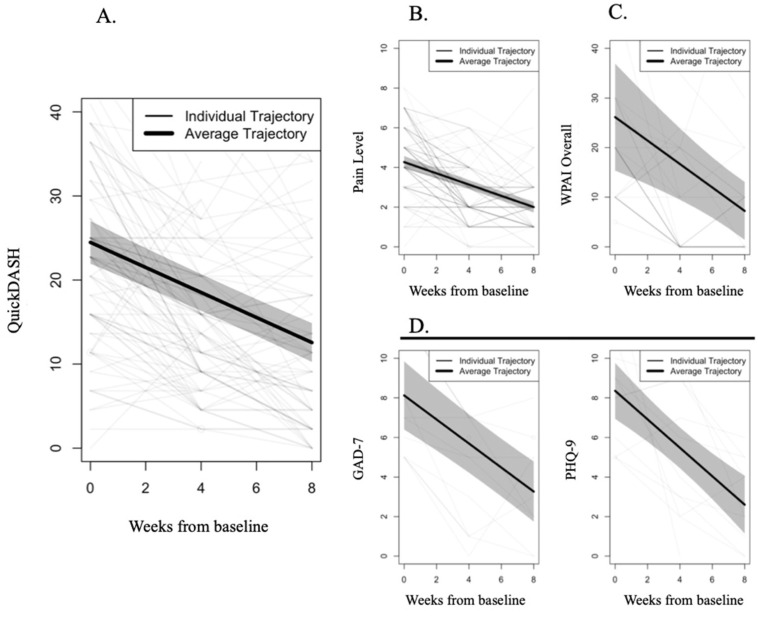
Longitudinal changes across time for all filtered variables (GAD-7 and PHQ-9 scores ≥ 5 points; WPAI scores > 0 points). (**A**). QuickDASH; (**B**). Pain Level; (**C**). WPAI overall; (**D**). Mental health: left—GAD-7; right—PHQ-9. Individual trajectories are depicted in lighter lines (with darker lines meaning overlap of trajectories), while average trajectories are depicted in bold lines, with shadowing representing 95% confidence intervals.

Participants reported an average reduction of 11.9 points (95% CI 9.8; 14.0) at the end of the program (mean 48.7% reduction, Table 2, Figure 3). Regarding the impact of covariates on trajectories, older participants and females reported higher levels of disability at baseline (Supplementary Table S3), with females presenting a steeper pace of improvement (−0.59, *p* = 0.036).

Among completers, 47.7% (41/86, *p* = 0.666) and 75.3% (64/85, *p* < 0.001) subjects reached the defined thresholds as response to treatment (responders) for 12 points reduction and 30% reduction, respectively. The estimated odds ratio (OR) for being a responder was 0.9 (95% CI 0.6; 1.4) and 3.1 (95% CI 1.1; 2.6), respectively, and not affected by any covariate (*p* > 0.05) (Supplementary Table S4).

#### 3.2.2. Secondary Outcomes

##### Pain

Participants reported moderate pain at baseline (4.3, 95% CI 4.0; 4.6), which was reduced by an average 2.3 points at program end, corresponding to an average change of 53.1% (Table 2, Figure 3). Similarly to the disability outcome, females showed a faster pace of recovery compared to males (−0.10, *p* = 0.034) despite the similar baseline levels (Supplementary Table S3). Pain reduction correlated with the improvement in disability (QuickDASH) (r(86) = 0.542, *p* < 0.001).

##### Analgesics Consumption

At baseline, approximately one-third of participants (41/132) were taking analgesics, while only 15.7% (14/89) continued taking analgesics at the study end.

##### Surgery Intent

Reported intention to undergo surgery was very low at baseline (mean 3.7, 95% CI 2.0; 5.4). Among those with baseline scores above 0, a significant average change of 4.4 points (95% CI 0.4; 8.4) was observed at the end of the study, corresponding to an overall decrease of 57.5%. Covariates showed no impact on recovery pace (*p* > 0.05).

##### Mental Health

Among participants who reported at least mild anxiety and depression symptomatology, a significant improvement was observed in both outcomes, representing a mean reduction of 4.9 points (95% CI 3.5; 6.2) in anxiety and 5.8 points (95% CI 3.7; 7.8) in depression at program end. This corresponds to overall reductions of 59.8% and 68.9% for anxiety and depression, respectively. Covariates did not impact these outcome trajectories, despite participants with higher BMI levels reporting less anxiety at baseline (*p* = 0.004). Anxiety reduction correlated with decrease in surgery intention (r(89) = 0.220, *p* = 0.038).

A significant mean reduction of 4.2 points, (95% CI 3.0; 5.3) (34.2% change) was observed in fear–avoidance beliefs (FAB), which correlated with disability (QuickDASH) improvement (r(89) = 0.219, *p* = 0.050).

##### Work Productivity

The reported low absenteeism at program start—4% (4/117)—precluded analysis through LGCA. Descriptively, this was further reduced to 2.4% by program end (2/82). Those with work productivity impairment at baseline attained a significant improvement of 75.6% average change in presenteeism (WPAI work: 18.4, 95% CI 11.5; 25.3).

For overall WPAI, a 72.3% improvement was observed (overall change 18.9 points, 95% CI 9.2; 28.6) at program end. Older participants reported higher baseline levels of WPAI overall (*p* = 0.012), but trajectories revealed a steeper recovery pace (−0.17, *p* = 0.006). WPAI overall improvement correlated with the reductions observed in disability (QuickDASH) (r(74) = 0.401, *p* < 0.001) and in FAB (r(73) = 0.310, *p* = 0.008).

Regarding non-work related activities impairment, a 67.5% improvement was observed (WPAI activity: 20.3, 95% CI 16.1; 24.3) which correlated with reductions in QuickDASH (r(86) = 0.443, *p* < 0.001) and pain (r(89) = 0.370, *p* < 0.001).

##### Engagement and Usability-Related Outcomes

Participants completed on average 25.6 (SD 11.6) exercise sessions, at a frequency of 3.5 (SD 1.4) sessions per week, corresponding to 313.5 (SD 146.9) minutes of total exercise time. Highly compliant individuals (i.e., performing an average of at least 4 sessions per week) reported greater change in the primary outcome QuickDASH (13.9 (11.1) vs. 11.6 (12.2)) than less engaged individuals, despite not reaching statistical significance (*p* = 0.200). Participants read an average of 3.7 (5.5) educational articles and interacted with PTs through the chat function, on average, 7.5 times (10.3). DCP-related participant satisfaction was 8.5 (1.6).

## 4. Discussion

### 4.1. Main Findings

Significant improvements were observed across all outcome measures, which benefited from very high engagement and completion rates attained in this multimodal DCP. A clinically meaningful improvement in disability (primary outcome) of 48.7% was observed, with 47.7% and 75.3% participants responding to treatment depending on the chosen MCIC for QuickDASH (12.0 points or 30% change, respectively). Importantly, this recovery was correlated with improvements in pain (53.1%), fear–avoidance beliefs (FAB) (34.2%) and both overall productivity (72.3%) and activities of daily living (67.5%). Additionally, significant improvements in analgesic consumption (from 30.4% at baseline to 15.7% at program end), intention to proceed to surgery (57.5%) and mental health (59.8% and 68.9% for anxiety and depression, respectively) were observed.

### 4.2. Comparison with Literature

Most reported clinical trials in conventional exercise-based therapy are focused on lateral elbow tendinopathy [72,73,74,75,76,77,78,79,80,81,82,83], with a growing number of studies specifically investigating home-based exercise interventions [74,75,77,78,79,81,82]. Our study included a real-world cohort with diversified EP conditions, whose demographics reflect the previously reported: higher prevalence of elbow tendinopathies, at working ages [3,13], with manual workers at increased risk [11,13,14,15,16]. Comparison to conservative intervention studies is hampered by significant differences in eligibility criteria, etiologies, outcome measures and baseline disability levels, with all of them focusing on patients with higher baseline disease burden and excluding patients with concomitant neck/thoracic/upper limb dysfunctions or neuropathies [74,75,76,77,78,79,81,82]. Overall evidence supports exercise effectiveness in EP management, despite disability improvement varying widely between studies [74,75,76,78,79,80,83]. Disability levels at the end of this program (12.56, 95% CI 10.25; 14.87) were within the ranges reported in previous studies (9–23 points), using either QuickDASH, DASH or the Patient-Rated Tennis Elbow Evaluation questionnaire (PRTEE) [74,75,78,79]. The difference between response rates derived by the two chosen cut-offs may be explained by a possible flooring effect exerted by an absolute MCIC in samples with lower baseline disabilities.

To the best of our knowledge, only one study addressed telerehabilitation of patients with EP. In this pilot study (N = 18), telerehabilitation was compared to conventional therapy in patients who underwent surgery after an elbow fracture [40]. Similarly to our DCP, this intervention consisted of asynchronous rehabilitation using a software device with biofeedback. No differences were observed between groups, supporting the feasibility and potential of telerehabilitation in the management of EP conditions. Our cohort was mostly composed of patients with elbow tendinopathies (75.8%), therefore precluding direct comparison with the aforementioned study.

In the present study, disability improvement was correlated with pain reduction (53.1%), which is in line with that reported in previous clinical trials (29–56.6%) [73,74]. Importantly, the overall pain change at the end of the program (2.3 points, 95% CI 1.9; 2.6) exceeds the minimal clinically important change of 2.0 points proposed by IMMPACT guidelines [84]. Given the disability and pain improvement results, it was not surprising to observe a marked reduction of participants’ intention to undergo surgery (57.5%). This finding is consistent with the recommendation of prioritizing conservative treatments, considering exercise potential to reduce the need for surgery [85,86] and avoid associated risks [3,4,19,20]. Evidence shows that a patient’s willingness to undergo surgery is one of the main predictors of future surgery [87], and individuals ending interventions without surgery intention are unlikely to proceed to surgery [88].

An increasing body of research support the importance of addressing mental health in MSK conditions [89,90,91,92]. Despite exerting an important influence on EP outcomes [9,10,93], this domain remains poorly explored. In the present study, among patients with at least mild baseline anxiety or depression, a significant improvement was observed at the end of the program (59.8% in GAD-7 and 68.9% in PHQ-9) which might reflect the importance of the biopsychosocial framework of the DCP. A combined intervention may also be instrumental in addressing FAB, which is consistently associated with a poor prognosis [60,94,95,96].

Although absenteeism was rarely reported, similarly to a prior population-based study [3], presenteeism was commonplace. Despite EP conditions’ strong impact on productivity [5,7], this domain is still unexplored after intervention studies. In the present study, a marked recovery of ~73% on productivity impairment (WPAI overall and work) was estimated in those reporting condition-related work limitations.

### 4.3. Patient Engagement

Most studies fail to report patient’s engagement metrics, particularly important in unsupervised home-based programs, which typically have the lowest adherence [97]. The observed high compliance and high completion rate strongly support an asynchronous digital care model, with results similar to those previously reported in RCTs evaluating conventional therapies for EP [74,76,77]. The high observed engagement might result from regular patient follow-up by the PT, which builds on the convenience of a completely-remote intervention. This is further supported by the high participant satisfaction scores (mean 8.5/10 (1.6)).

### 4.4. Strengths, Limitations, and Future Studies Recommendations

The study strengths include the large sample size derived from real-world conditions, along with the novelty of a modality still scarcely explored in patients with EP conditions. The DCP described in the present study followed a multimodal evidence-based approach within the context of a biopsychosocial framework, using innovative technology to allow real-time biofeedback and asynchronous, continuous monitoring with regular communication. A previous study reported that some patients perceived the feedback from motion sensor technologies to be more accurate than from a therapist [37]. Other strengths include the assessment of multiple outcome domains through validated and widely used measures, and the inclusion of a diverse EP cohort from various geographical locations.

The statistical methodologies took into account the inclusion of real-world data, providing a measure of fitness, offering the transparency that is lacking in most literature studies, and handling missing data through FIML, a method robust to attrition bias, which acknowledges that repeated measures on the same individual are correlated.

Limitations are mainly related to the study design—a single-arm open-label study with no control or comparator group. However, this study focused on an exploratory analysis of real-world data to support further research. Considering the high accessibility of this DCP, using a wait-and-see control group would not be practical and might raise ethical concerns. Moreover, the study design precluded the individual assessment of each component of the multimodal intervention, and long-term follow-up was not carried out. These limitations should be considered in the planning of future studies.

## 5. Conclusions

The observed high engagement, completion, and satisfaction rates reveal patient acceptance of this care delivery mode. Clinically meaningful improvements in pain and disability were observed, which were accompanied by reduced analgesic usage and surgery intent, and enhanced mental health and work productivity, in line with current literature. Digital interventions may have great potential to manage EP conditions, benefiting patients and society alike.

## Figures and Tables

**Table 1:** Baseline characteristics of study participants (N = 132).

Characteristic	Entire Cohort
Age (years), mean (SD)	51.3 (9.9)
Age categories (years), N (%):	
<25	0 (0.0)
25–40	20 (15.2)
40–60	89 (67.4)
>60	23 (17.4)
Sex, N (%)	
Female	115 (60.8)
Male	73 (38.6)
BMI, mean (SD)	26.8 (5.3)
BMI categories, N (%):	
Underweight (<18.5)	0 (0.0)
Normal (18.5–25)	62 (47.0)
Overweight (25–30)	38 (28.8)
Obese (30–40)	29 (22.0)
Morbidly obese (>40)	3 (2.3)
Laterality	
Left	37 (28.0)
Right	88 (66.7)
Bilateral	7 (5.3)
Elbow pain-related condition, N (%):	
Tendinopathies	100 (75.8)
Lateral elbow tendinopathy	66 (50.0)
Medial elbow tendinopathy	26 (19.7)
Other tendinopathies	8 (6.1)
Elbow pain after non-traumatic injury	9 (6.8)
Elbow pain after traumatic injury	6 (4.5)
Distal nerve entrapment neuropathy	11 (8.3)
Non-specific	6 (4.6)
Pain duration, N (%):	
Acute (<12 weeks)	72 (54.5)
0–4 weeks	12 (9)
4–12 weeks	60 (45)
Chronic (>12 weeks)	60 (45.5)
<6 months	29 (22)
6–12 months	19 (14)
≥1 year	12 (10)
Employment status, N (%):	
Employed (part-time or full-time)	123 (93.2)
Unemployed (not working or retired)	9 (6.8)
Occupation type, N (%):	
White collar	68 (51.5)
Blue collar	40 (30.3)
Other (e.g., retired)	19 (14.4)
Not available	5 (3.8)

Abbreviations: BMI, body mass index.

**Table 2:** Outcome changes between baseline and 8-weeks: intent-to-treat approach (unconditional model).

Outcome, Mean (95% CI)	N	Baseline	End-Of-Program	Mean Change	% Change
QuickDASH	130	24.48 (21.94; 27.01)	12.56 (10.25; 14.87)	11.92 (9.80; 14.04)	48.7%
Pain Level	132	4.27 (3.96; 4.57)	2.00 (1.73; 2.27)	2.27 (1.92; 2.61)	53.1%
Surgery Intent > 0	50	7.58 (2.83; 12.33)	3.22 (1.62; 4.83)	4.36 (0.37; 8.35)	57.5%
Surgery Intent	132	3.70 (1.96; 5.44)	1.34 (0.61; 2.06)	2.36 (0.84; 3.89)	63.9%
FABQ-PA	132	12.21 (11.28; 13.15)	8.03 (6.82; 9.24)	4.18 (3.02; 5.34)	34.2%
GAD-7 ≥ 5	16	8.12 (6.40; 9.85)	3.26 (1.74; 4.79)	4.86 (3.49; 6.23)	59.8%
GAD-7	132	1.87 (1.37; 2.37)	1.28 (0.83; 1.74)	0.59 (0.12; 1.05)	31.4%
PHQ-9 ≥ 5	19	8.36 (6.94; 9.77)	2.60 (1.14; 4.06)	5.76 (3.74; 7.77)	68.9%
PHQ-9	132	1.86 (1.32; 2.41)	0.99 (0.56; 1.43)	0.87 (0.28; 1.46)	46.7%
WPAI Overall > 0	46	26.14 (15.36; 36.92)	7.24 (1.39; 13.08)	18.90 (9.20; 28.60)	72.3%
WPAI Overall	117	7.74 (4.31; 11.16)	3.71 (1.99; 5.43)	4.03 (1.32; 6.74)	52.1%
WPAI Work > 0	45	24.34 (16.15; 32.53)	5.94 (0.83; 11.04)	18.40 (11.52; 25.28)	75.6%
WPAI Work	117	7.50 (4.51; 10.49)	3.31 (1.69; 4.94)	4.19 (1.81; 6.57)	55.8%
WPAI Activity > 0	104	30.08 (26.06; 34.11)	9.78 (6.30; 13.25)	20.30 (16.14; 24.27)	67.5%
WPAI Activity	132	23.03 (18.98; 27.08)	8.77 (5.92; 11.62)	14.26 (10.46; 18.07)	61.9%

Analyses were performed both for unfiltered cases and filtering for above zero (>0) for surgery intent (individuals with intention to undergo surgery at baseline) and WPAI (individuals with productivity impairment at baseline); and above or equal to five (≥5) points for GAD-7 and PHQ-9 (individuals with at least mild anxiety and depression at baseline). Abbreviations: QuickDASH, Quick Disabilities of the Arm, Shoulder and Hand questionnaire; FABQ-PA, Fear-Avoidance Beliefs Questionnaire for physical activity; GAD-7, Generalized Anxiety Disorder 7-item scale; PHQ-9, Patient Health 9-item questionnaire; WPAI, Work Productivity and Activity Impairment questionnaire.

## Data Availability

The data presented in this study are available on request from the corresponding author. The data are not publicly available due to privacy restrictions.

## Supplementary Materials

The following supporting information can be downloaded at: https://www.mdpi.com/article/10.3390/ijerph19159198/s1, Figure S1: Example path diagram for the LGC models used in the current study; Table S1: Baseline characteristics of completers vs. non-completers; Table S2: Unconditional latent growth curve analysis: intent-to-treat; Table S3: Intent-to-treat conditional latent growth curve model, with body mass index, age, sex, and acuity as covariates; Table S4: (A) Responder analysis for primary outcome (QuickDASH) considering an absolute MCIC of 12.0 and a relative MCIC of 30% of change; (B) association of baseline variables with odds of a successful outcome (responder).
