# Supplementary material for: Digital Rehabilitation for Elbow Pain Musculoskeletal Conditions: A Prospective Longitudinal Cohort Study
Source: Int J Environ Res Public Health. 2022 Jul 27;19(15):9198. doi: 10.3390/ijerph19159198 (PMC9367806; doi:10.3390/ijerph19159198)
Supplement: Supplementary file 1 [file ijerph-19-09198-s001.zip › ijerph-1797624-supplementary.pdf]

Supplementary Materials

Digital rehabilitation for elbow pain musculoskeletal conditions: a prospective longitudinal cohort study

Dora Janela, Fabíola Costa, Maria Molinos, Robert G. Moulder, Jorge Lains, Virgílio Bento, Justin K Scheer, Vijay Yanamadala, Steven P. Cohen, Fernando Dias Correia

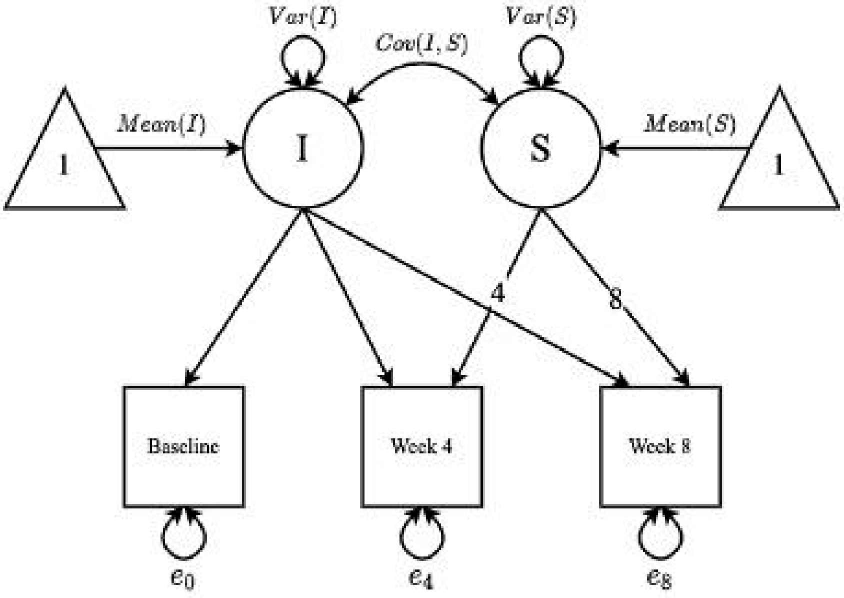

Supplementary Figure S1. Example path diagram for the LGC models used in the current study. LGCs are a form of structural equation model for modeling longitudinal processes. Squares represent outcome variables at baseline, 4 weeks, and 8 weeks. Circles represent average latent intercept (I), and slope (S) components across all participants. Single-headed arrows represent fixed loadings relating the outcome variables to the latent components. Double-headed arrows represent either variance, or covariance.

Latent growth curve (LGC) models take the form:

$$Y_{ij} = I + St_i + u_{Ij} + u_{sj}t_i + e_{ij}$$

where Y is the outcome score for person, j, at time, i, t_i is a vector of time points representing [0, 4, 8] weeks, I is an intercept term representing the average value at t=0 for all participants, S is a slope term representing the average linear change of Y_{ij} over time for all participants, u_{Ij} is a random intercept term representing individual participant variation in I, u_{sj} is a random slope term representing individual participant variation in S, and e_{ij} is an error term.

Supplementary Table S1. Baseline characteristics of completers vs non-completers.

Characteristic	Completers (N=112)	Non-completers (N=20)	p
Age (years), mean (SD)	51.5 (9.9)	50.1 (9.7)	0.284
Age categories, N (%):			0.343
<25	0 (0.0)	0 (0.0)	
25-40	15 (13.4)	5 (25.0)	
40-60	78 (69.6)	11 (55.0)	
> 60	19 (17.0)	4 (20.0)	
Sex, N (%):			0.941
Female	57 (50.9)	10 (50.0)	
Male	55 (49.1)	10 (50.0)	
BMI, mean (SD)	27.0 (5.5)	26.0 (4.4)	0.224
BMI categories, N (%):			0.822
Underweight (<18.5)	0 (0.0)	0 (0.0)	
Normal (18.5-25)	53 (47.3)	9 (45.0)	
Overweight (25-30)	31 (27.7)	7 (35.0)	
Obese (30-40)	25 (22.3)	4 (20.0)	
Morbidly obese (>40)	3 (2.7)	0 (0.0)	
Employment status, N (%):			0.726
Employed (part-time or full-time)	104 (92.9)	19 (95.0)	
Unemployed	8 (7.1)	1 (5.0)	
Occupation type, N (%):			0.379
White collar	59 (52.7)	9 (45.0)	
Blue collar	31 (27.7)	9 (45.0)	
Other (e.g. retired)	17 (15.2)	2 (10.0)	
Not available	5 (4.5)	0 (0.0)	
Laterality, N (%):			0.752
Right	76 (67.9)	12 (60.0)	
Left	30 (26.8)	7 (35.0)	
Bilateral	6 (5.4)	1 (5.0)	
Elbow pain-related condition, N (%):			**.014**
Tendinopathies	86 (76.9)	14 (70.0)	
Lateral elbow tendinopathy	61 (54.5)	5 (25.0)	
Medial elbow tendinopathy	19 (17.0)	7 (35.0)	
Other tendinopathies	6 (5.4)	2 (10.0)	
Elbow pain after non-traumatic injury	9 (8.0)	0 (0.0)	
Elbow pain after traumatic injury	6 (5.4)	0 (0.0)	
Distal nerve entrapment neuropathy	8 (7.1)	3 (15.0)	
Non-specific	3 (2.7)	3 (15.0)	
Pain duration, N (%):			.658
Acute (<12 weeks)	62 (55.4)	10 (50.0)	
0-4 weeks	11 (9.8)	1 (5.0)	
4-12 weeks	51 (45.6)	9 (45)	
Chronic (>12 weeks)	50 (44.6)	10 (50.0)	
<6 months	22 (19.6)	7 (35.0)	
6-12 months	17 (15.2)	2 (10.0)	
≥1 year	11 (9.8)	1 (5.0)	
**Outcome measures, mean (SD)**			
Pain Level	4.5 (1.8)	4.4 (1.9)	0.455
QuickDASH	25.9 (13.6)	23.1 (14.6)	0.198
Analgesics, N (%)	34 (30.4)	7 (35.0)	0.679
Surgery Intent	5.7 (12.2)	4.7 (11.8)	0.364
FABQ-PA	12.3 (5.7)	13.7 (4.9)	0.147
GAD-7	1.8 (2.9)	1.6 (3.2)	0.411
PHQ-9	2.0 (3.4)	1.2 (1.5)	**0.041**
WPAI Overall	12.2 (20.4)	3.4 (7.5)	**<.001**
WPAI Work	10.6 (17.3)	3.4 (7.5)	0.002
WPAI Time	1.9 (11.5)	0.0 (0.0)	0.235
WPAI Activities	25.2 (21.1)	19.5 (20.4)	0.134

Abbreviations: BMI, Body mass index; QuickDASH, Quick Disabilities of the Arm, Shoulder and Hand questionnaire; FABQ-PA, Fear-Avoidance Beliefs Questionnaire for physical activity; GAD-7, Generalized Anxiety Disorder 7-item scale; PHQ-9, Patient Health 9-item questionnaire; WPAI, Work Productivity and Activity Impairment questionnaire. Note: Significant p-values are presented in bol

Supplementary Table S2. Unconditional Latent Growth Curve analysis: intent-to-treat.

		Intercept		Slope		Fit				
**Outcome**	**N**	**Mean (SD)**	***p***	**Mean (SD)**	***p***	**Chi-sq (df)**	***p***	**RMSEA**	**CFI**	**SRMR**
QuickDASH	130	24.48 (10.79)	**< .001**	-1.49 (0.57)	**< .001**	10.82 (1)	0.001	0.273	**0.92**	0.062
Pain Level	132	4.27 (0.78)	**< .001**	-0.28 (0.03)	**< .001**	15.06 (1)	< .001	0.326	0.47	0.113
Surgery Intent >0	50	7.58 (0.03)	**0.002**	-0.54 (0.03)	**0.032**	20.49 (1)	< .001	0.624	0.00	0.236
Surgery Intent	132	3.70 (5.26)	**< .001**	-0.30 (0.03)	**0.002**	16.41 (1)	< .001	0.342	0.63	0.134
FABQ-PA	132	12.21 (3.52)	**< .001**	-0.52 (0.19)	**< .001**	4.52 (1)	0.034	0.163	**0.96**	**0.049**
GAD-7 ≥5	16	8.12 (3.52)	**< .001**	-0.61 (0.03)	**< .001**	6.58 (1)	0.010	0.591	0.40	0.222
GAD-7	132	1.87 (2.21)	**< .001**	-0.07 (0.11)	**0.013**	0.9 (1)	**0.343**	**0.000**	**1.00**	**0.018**
PHQ-9 ≥5	19	8.36 (0.03)	**< .001**	-0.72 (0.39)	**< .001**	1.4 (1)	**0.237**	0.144	0.84	0.120
PHQ-9	132	1.86 (2.93)	**< .001**	-0.11 (0.40)	**0.004**	1.11 (1)	**0.292**	**0.029**	**1.00**	**0.018**
WPAI Overall >0	46	26.14 (20.06)	**< .001**	-2.36 (2.71)	**< .001**	16.06 (1)	< .001	0.572	0.19	0.246
WPAI Overall	117	7.74 (12.26)	**< .001**	-0.50 (1.01)	**0.004**	7.86 (1)	0.005	0.235	0.87	0.066
WPAI Work >0	45	24.34 (17.29)	**< .001**	-2.30 (2.33)	**< .001**	11.71 (1)	< .001	0.488	0.32	0.209
WPAI Work	117	7.50 (10.80)	**< .001**	-0.52 (0.86)	**< .001**	5.81 (1)	0.016	0.197	**0.90**	0.058
WPAI Activity >0	104	30.08 (16.21)	**< .001**	-2.54 (1.44)	**< .001**	10.31 (1)	0.001	0.299	0.78	0.100
WPAI Activity	132	23.03 (16.22)	**< .001**	-1.78 (1.34)	**< .001**	7.61 (1)	0.006	0.224	0.89	0.066

Note: Significant p-values are presented in bold. Trajectories were calculated through intercept (i.e., initial estimated value at baseline), and slope (i.e. linear outcome change per week) for each variable.

Supplementary Table S3. Intent-to-treat Conditional Latent Growth Curve Model,with body mass index, age, sex and acuity as covariates.

	**Age**		**Female**		**BMI**		**Chronic**		**FIT**				
**Outcome**	**Intercept**	**Slope**	**Intercept**	**Slope**	**Intercept**	**Slope**	**Intercept**	**Slope**	**Chi-sq (df)**	***p***	**RMSEA**	**CFI**	**SRMR**
Quickdash	**0.26 (0.023)**	-0.02 (0.072)	**8.90 (< .001)**	**-0.59 (0.036)**	0.29 (0.175)	-0.01 (0.774)	0.60 (0.777)	-0.26 (0.365)	13.58 (4)	0.009	0.135	**0.93**	**0.04**
Pain Level	0.03 (0.088)	0 (0.053)	0.55 (0.057)	**-0.10 (0.034)**	-0.01 (0.609)	0 (0.626)	0.08 (0.775)	0.02 (0.731)	22.07 (4)	< .001	0.185	0.46	0.076
Surgery Intent >0	-0.01 (0.948)	0 (0.853)	-0.85 (0.780)	-0.18 (0.662)	0.16 (0.546)	-0.03 (0.447)	-1.20 (0.670)	0.23 (0.468)	25.63 (4)	< .001	0.329	0.00	0.159
Surgery Intent	-0.01 (0.935)	0 (0.741)	-2.16 (0.151)	0.2 (0.269)	-0.01 (0.881)	0 (0.751)	-1.18 (0.418)	0.24 (0.182)	18.56 (4)	< .001	0.166	0.61	0.084
FABQ-PA	-0.01 (0.783)	0 (0.893)	0.55 (0.559)	-0.03 (0.800)	-0.02 (0.773)	0.01 (0.457)	0.63 (0.513)	0.15 (0.291)	8.96 (4)	**0.062**	0.097	**0.93**	**0.038**
GAD-7 ≥5	-0.02 (0.838)	-0.02 (0.405)	1.67 (0.209)	-0.29 (0.257)	**-0.29 (0.004)**	0.04 (0.052)	2.31 (0.117)	0.18 (0.309)	18.41 (4)	0.001	0.474	0.31	0.259
GAD-7	0 (0.944)	0 (0.327)	**0.98 (0.043)**	-0.06 (0.325)	-0.01 (0.883)	0 (0.704)	0.87 (0.077)	-0.10 (0.108)	2.83 (4)	**0.587**	**0.000**	**1.00**	**0.017**
PHQ-9 ≥5	0.07 (0.429)	0 (0.966)	1.95 (0.157)	-0.16 (0.516)	-0.06 (0.759)	0 (0.900)	-1.18 (0.376)	0.39 (0.218)	6.64 (4)	**0.156**	0.186	0.03	0.107
PHQ-9	0 (0.874)	0 (0.733)	0.36 (0.527)	0.05 (0.564)	0.05 (0.293)	0 (0.825)	-0.25 (0.642)	-0.02 (0.767)	8.88 (4)	**0.064**	0.096	**0.95**	**0.034**
WPAI Overall >0	**0.71 (0.012)**	**-0.17 (0.006)**	-1.19 (0.847)	1.31 (0.181)	0.86 (0.096)	-0.06 (0.313)	-9.74 (0.056)	0.69 (0.354)	17.9 (4)	0.001	0.275	0.53	0.147
WPAI Overall	0.16 (0.189)	**-0.05 (0.027)**	1.98 (0.518)	0.22 (0.556)	0.24 (0.322)	-0.03 (0.273)	**-7.67 (0.006)**	0.55 (0.110)	11.92 (4)	0.018	0.122	0.87	0.046
WPAI Work >0	**0.48 (0.042)**	**-0.15 (0.010)**	2.34 (0.624)	1.16 (0.158)	0.98 (0.053)	-0.06 (0.314)	-5.88 (0.190)	-0.01 (0.989)	13.31 (4)	0.010	0.227	0.70	0.096
WPAI Work	0.10 (0.340)	**-0.04 (0.044)**	3.36 (0.255)	0.10 (0.767)	0.32 (0.201)	-0.04 (0.188)	-6.24 (0.015)	0.34 (0.310)	10.52 (4)	**0.033**	0.111	0.89	**0.046**
WPAI Activity >0	0 (0.990)	-0.02 (0.525)	4.47 (0.201)	-0.58 (0.238)	**1.07 (0.008)**	-0.11 (0.072)	-9.74 (0.056)	0.69 (0.354)	10.9 (4)	0.028	0.129	0.85	0.062
WPAI Activity	-0.09 (0.528)	0 (0.868)	4.06 (0.249)	-0.52 (0.270)	0.60 (0.109)	-0.08 (0.091)	-4.81 (0.164)	-0.10 (0.846)	8.64 (4)	**0.071**	0.094	**0.92**	**0.043**

Note: Significant p-values are presented in bold. Trajectories were calculated through intercept (i.e., initial estimated value at baseline), and slope (i.e. linear outcome change per week) for each variable. If a significant chi-square is found for a model, then CFI values > .9, or RMSEA values < .08, or SRMR values <.05 signify models with acceptable fit (presented in bold).

Supplementary Table S4. A. Responder analysis for primary outcome (QuickDASH) considering an absolute MCIC of 12.0 and a relative MCIC of 30% of change; B. Association of baseline variables with odds of a successful outcome (responder).

A.

MCIC	Responded	Did not Respond	Odds Ratio (Responded)	95% CI	Probability (Responded)	z	*p*
30%	64	21	3.05	1.90; 5.11	0.753	4.43	**<.001**
12.0	41	45	0.91	0.59; 1.39	0.477	-0.43	0.666

B.

Parameter	OR being a responder	95% CI	z	*p*
**30% MCIC**				
Intercept	1.71	1.35; 5.91	2.69	**0.007**
Age	0.99	0.94; 1.05	-0.21	0.834
BMI	0.98	0.90; 1.08	-0.42	0.677
Female	1.26	0.46; 3.41	0.45	0.651
**12.0 MCIC**				
Intercept	0.44	0.21; 0.87	-2.29	**0.022**
Age	1.04	0.99; 1.09	1.46	0.145
BMI	0.97	0.90; 1.05	-0.71	0.481
Female	3.33	1.37; 8.51	2.59	**0.010**
Chronic	1.69	0.66; 4.51	1.075	0.283

Abbreviations: BMI, Body mass index; MCIC, minimal clinically important changes
Note: Significant p-values are presented in bold.
